# Incidental Intraoperative Diagnosis of Term Conjoined Twins: A Case Series

**DOI:** 10.31729/jnma.7929

**Published:** 2023-01-31

**Authors:** Ravi Kumar Shah, Arushi Jaiswal, Rehana Mushtaq, Sana Ansari, Pravin Shah, Ruby Shrestha, Sneha Shakya, Saroj Yadav, Madhu Sah, Saruna Pathak, Jagat Prasad Deep

**Affiliations:** 1 Department of Obstetrics and Gynaecology, National Medical College and Teaching Hospital, Birgunj, Parsa, Nepal; 2 Department of Anaesthesia, National Medical College and Teaching Hospital, Birgunj, Parsa, Nepal

**Keywords:** *conjoined*, *monozygotic twinning*, *siamese twins*, *twins*

## Abstract

Conjoined twins (Siamese twins) represent the rarest form of twin pregnancy. Reported here are two rare cases of conjoined term twins presented to the department of Obstetrics and Gynaecology within 3 months. The first case, 32 years of gravida 6 parity 5 referred from the periphery on 16 May 2022 after a full trial of labour following multi-organ dysfunction and term intrauterine dead twins. Intraoperatively it was dead conjoined thoraco-omphalopagus females. The patient died after 3 days following multiorgan dysfunction syndrome and disseminated intravascular coagulation. The second case, 22 years gravida 2 parity 1 also referred from periphery on 8 August 2022 in the second stage of labour with the diagnosis of 39 weeks intrauterine dead twins with obstructed labour, delivered by caesarean with intraoperative conjoined dead females of thoracophagus type. Twins is itself a highrisk pregnancy and this rare diagnosis with complications could have been prevented by regular antenatal checkups, ultrasonography performed by radiologists and early referral antenatally in labour along with a multidisciplinary approach.

## INTRODUCTION

Conjoined twins (Siamese twins) represent the rarest form of twin pregnancy.^1^ It refers to monozygotic twins that are physically fused in utero due to incomplete embryonic division which occurs 13^th^ to 15^th^ day following conception resulting in different degrees of fusion defects between two fetuses.^2,3^ The incidence ranges from 1 in 49,000 births to 1 in 189,000 births, with a somewhat higher incidence in Southeast Asia, Africa and Brazil.^4^ Approximately half are stillborn, and an additional one-third die within 24 hours. Most live births are female,^5^ with a ratio of 3:1.^6^

## CASE REPORT

### CASE 1

**Figure 1:**
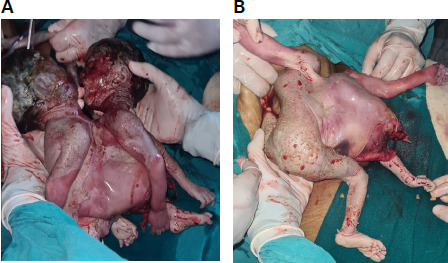
A) Conjoined twin - thoraco- omphalopagus type, B) Conjoined twin - detached one lower limb.

A 32 years old unbooked grand multipara was referred from the peripheral birth centre to our tertiary care hospital on 16 May 2022 with diagnosis gravida 6 parity 5 living 5 at term pregnancy with the full trial of labour with multiorgan dysfunction syndrome and intrauterine fetal death (IUFD). On history, in her antenatal period, she visited only twice to a district hospital where her second-trimester scan showed intrauterine alive twin pregnancy of diamniotic dichorionic type. The patient presented us with the complaint of generalised body swelling for 1.5 months, pain abdomen, headache, vomiting, epigastric pain for 7 days and decreased fetal movement for 4 days. Here, on presentation, she was ill looking, pale and tachycardic with gross bilateral pedal and anterior abdominal oedema. On per abdomen, the lie is longitudinal, the height of the fundus was at term, two mild contractions along with multiple fetal parts palpable and fetal heart sound audible. On per vaginal examination, the vulva vagina was soaked with blood, the cervical os is fully dilated, and completely effaced, and the broad irregular fetal structure felt well above the ischial spine (according to the visitor, one lower limb was already detached in the process of labour). Blood investigation came out deranged suggestive of multiorgan dysfunction syndrome. A plan of caesarean section was made for a maternal indication with high risk consent. Lower section caesarean section was performed on 17 May 2022 with the following intraoperative findings noted incidentally. Intraperitoneal moderate amount of straw coloured ascitic fluid; bladder-edematous; dead thoraco-omphalopagus type of conjoined twin; placenta-anterior (monochorionic, monoamniotic); bilateral tubes and ovaries-normal (tubal ligation done); estimated blood loss-750 ml and urine output-250 ml (haemorrhagic). Fetal outcome: dead conjoined twin of thoraco-omphalopagus type; both female; weight-4.5 kg. Examination of conjoined twins showed: two heads with a normal face, fused thorax and abdomen, four upper limbs and three lower limbs (corresponding to the history given by the visitors. The patient died after 3 days of ICU stay following multiorgan dysfunction syndrome and disseminated intravascular coagulation (DIC) (Figure 1).

### CASE 2

**Figure 2:**
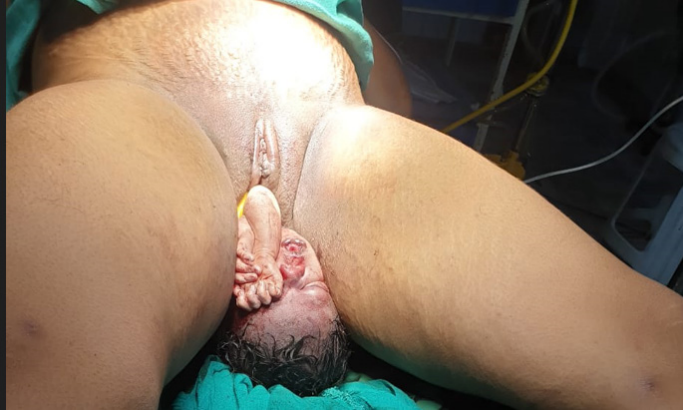
Fetal head stuck outside the vaginal introitus.

A 22 years old woman presented on 8 August 2022 in the second stage of labour with a diagnosis of gravida 2 parity 1 living 1 at 39 weeks with IUFD twin with obstructed labour. She was referred after a full trial of labour and had no prenatal visits, presented with abdominal pain and not perceiving fetal movement for 1 day. On examination, her general condition was ill looking and stable vitals. Abdominal examination showed 36 weeks sized uterus, 2 moderate contractions and fetal heart sound was not audible. The fetal head was stuck outside the vaginal introitus with no signs of life (Figure 2).

**Figure 3:**
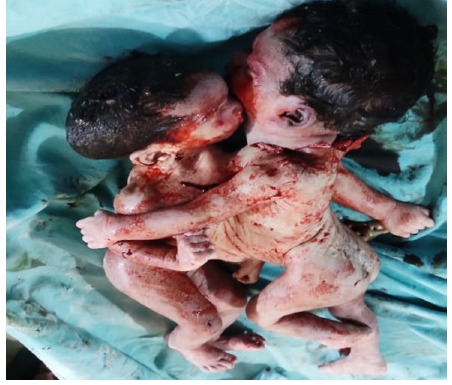
Conjoined twin - Thoracophagus type.

Per vaginal examination revealed some sort of fixed obstruction making the susceptibility of conjoined twin. The lab reports revealed anaemic and other investigations within the normal range (complete blood count, renal function test, liver function test, bleeding profile). The decision of caesarean section was made after taking high risk consent including a hysterectomy. The caesarean section was performed under general anaesthesia. A lower segment caesarean section was performed, leading to findings of conjoined with the following operative findings. Lower uterine segment-well formed; bladder-edematous; presentation-dead conjoined twin of thoracophagus type; breech extraction performed; placenta-anterior (monochorionic, monoamniotic); bilateral tubes and ovaries-normal; estimated blood loss-500-600 ml and urine output-50 ml (haemorrhagic). Fetal outcome: dead conjoined twin of thoracophagus type; both female; weight-4.5 kg. Examination of conjoined twins showed: two heads with a normal face, fused thorax and, four limbs (2 upper and lower) The postoperative period was uneventful and discharged after 7 days (Figure 3).

## DISCUSSION

A serious and uncommon complication of monochorionic twins is conjoined twins. After day 13 of fertilization, cleavage or axis duplication results in conjoined twinning. It is not clear how conjoined twins occur. Both the fission theory and fusion theory are well known.^7,8^ The fission theory suggests that the embryo undergoes incomplete division 13-15 days after fertilization, resulting in conjoined twins. The fusion theory suggests that two separate embryos undergo a second fusion 13 days after fertilization. The types of conjoined twins and their frequency are as follows:28% were thoraco-omphalopagus (joined at the thorax and abdomen), 18.5% were thoracopagus (joined at the thorax), 10% were omphalopagus (joined at the abdomen), 10% were heteropagus (parasitic twins), and 6% were craniopagus (joined at the level of the cranium). The pyopagus (joined at the sacrum and perineum), rachipagus (joined at the vertebral column), ischiopagus (joined at the lower abdomen and pelvis), and cephalopagus (joined from the head to the umbilicus) are examples of less frequently observed conjoined twins.^9^ For separation surgery, the location of the fusion and the organs involved are primary considerations. Typically, 25% of live births live long enough to be candidates for surgery.^7^ Conjoined twins are a rare condition that is uniquely identifiable on prenatal ultrasonography. The prognosis for conjoined twins is generally poor. The survival rate is 7.5% overall and 60% of surgically separated patients survive.^10^ Ultrasonography in the first trimester remains the most effective method for early pregnancy diagnosis. Additionally, prenatal magnetic resonance imaging can assist in determining the kind of conjunction, identifying embryological anomalies, and characterizing tissue. Modern techniques like 3D printing may assist with surgical pre-planning and subsequent separation, if necessary.^11^ The approach to manages conjoined twins is complex. Conjoined twins can be separated into the following groups with regard to prognosis: those who do not survive in utero, those who survive pregnancy but do not survive past infancy, those who survive infancy, but cannot be separated and those who survive infancy and can be surgically separated. Management and prognosis are so complicated, family prenatal counselling is very important.^12^ Prenatal ultrasound diagnoses of conjoined twins are based on these characteristics:^6,12^ (1) a single placenta without an amniotic septum; (2) fetuses lying in the same constant position with their heads and body parts always at the same level; (3) inseparable body and skin contours; (4) fetuses facing each other with hyperflexion of the cervical spines, sharing of organs, and a single umbilical cord with more than three vessels; (5) fewer limbs in some conjoined twins than in normal twins and (6) abnormal flexion of the spine. If necessary, MRI can be used to aid in diagnosis.^13^

Conjoined twins are more common in females with thoraco-omphalopagus followed by thoracophagus type which is similar to this case report. The regular antenatal checkups and ultrasonography performed by specialists following an early diagnosis of this rare complication may have prevented both women from becoming pregnant to term with complications. Termination could have been planned if a diagnosis was made early. Both cases presented complications late in labour; however, if they had arrived at the tertiary centre earlier, the mode of treatment could have been altered by surgically separating the fetus based on attachment. Despite the fact that we encounter numerous complications, this kind of uncommon complication is rare these days thanks to technological advancements. Our centre reported two cases of incidental diagnosis of conjoined twins within three months; however, many other patients do not even visit the tertiary centre, so no cases are reported. Early ultrasound diagnosis and awareness of rare complications are essential for obstetricians and ultrasound specialists. In these instances, the mode of treatment would have changed with prompt diagnosis and referral to a tertiary centre. However, in both instances, complications were discovered during late in labour due to inadequate awareness, resources, and antenatal checkups, resulting in the incidental intraoperative discovery of term conjoined twins with one maternal mortality and intrauterine fetal death status.
